# Sexual Dimorphism of ZEB1 Expression and Function in Glioblastoma

**DOI:** 10.3390/cells15151428

**Published:** 2026-08-06

**Authors:** Ben E. Whittaker, Samuel Davies, Jeffrey C. F. Kwan, Annabelle Gordon-Smith, Florian A. Siebzehnrubl

**Affiliations:** European Cancer Stem Cell Research Institute, School of Biosciences, Cardiff University, Cardiff CF10 3AX, UK

**Keywords:** Brain cancer, gene regulation, transcriptomics

## Abstract

**Highlights:**

**What are the main findings?**

- High ZEB1 expression predicts a survival advantage only in female glioblastoma patients in the TCGA-GBM cohort.
- Sex-stratified transcriptomic and qPCR analyses identify Y-linked chromatin modifiers KDM5D and UTY as ZEB1-regulated candidates that distinguish ZEB1-high male from female tumors.

**What are the implications of the main findings?**

- ZEB1’s prognostic value in glioblastoma depends on patient sex, indicating that biomarker development and risk stratification should explicitly incorporate sex as a biological variable.
- The linkage between ZEB1 and sex-chromosome-encoded epigenetic regulators suggests that sex-specific chromatin states may underlie distinct therapeutic vulnerabilities in male versus female GBM.

**Abstract:**

Glioblastoma (GBM) exhibits marked sex differences in incidence, outcome, and molecular regulation, yet the mechanisms underlying these disparities remain poorly defined. ZEB1 is a neurodevelopmental transcription factor implicated in GBM progression and cellular plasticity, but its prognostic and functional relevance may differ by sex. Here, we analyzed TCGA-GBM transcriptomic and clinical data to assess the relationship between ZEB1 expression, patient sex, and survival and to identify sex-specific transcriptional programs associated with ZEB1. Patients were stratified by ZEB1 expression and sex, followed by differential expression analysis, functional enrichment, and survival modeling. High ZEB1 expression was associated with improved overall survival in female patients but not in male patients. Sex-stratified transcriptomic analysis revealed distinct ZEB1-associated gene expression signatures, with enrichment of chromatin-modifying and demethylase-related pathways among male–female comparisons. Candidate Y-linked epigenetic regulators, including KDM5D and UTY, were differentially expressed in ZEB1-high male tumors. qPCR validation in male and female patient-derived GBM cell lines supported sex-dependent regulation of these candidates and showed that KDM5D and UTY expression was reduced following ZEB1 knockdown in male cells. Together, these findings identify a sex-dependent prognostic role for ZEB1 in GBM and suggest that ZEB1 interacts with sex-chromosome-linked epigenetic regulators to shape tumor transcriptional states.

## 1. Introduction

IDH-wildtype diffuse astrocytoma WHO grade 4 (glioblastoma, GBM) is the most common and aggressive primary malignant brain tumor in adults, with a higher incidence in males [1]. In one large cohort of GBM patients, median survival was approximately 20 months in female patients and 17.5 months in males, suggesting a female survival advantage that is modest but reproducible [2,3]. In the 2021 WHO classification of CNS tumors, IDH-mutant astrocytomas are no longer categorized as glioblastoma (IDH-wildtype), and survival estimates—including observed sex differences—may vary depending on whether historical cohorts include or exclude IDH-mutant grade 4 astrocytomas under the GBM label. While sex-specific differences in GBM biology and therapy response are increasingly recognized in the literature [3,4,5], the molecular drivers underlying sex biases in GBM are incompletely understood. Understanding of GBM sex bias is impeded by the profound heterogeneity at multiple biological levels, which also represents a major obstacle to effective therapy.

Transcriptional heterogeneity in GBM has led to the identification of different molecular subclasses [6] and cellular states [7] that coexist within the same tumors, are influenced by the local microenvironment [8], and which also show sex-specific differences [3]. GBM transcriptional programs partially map onto neurodevelopmental trajectories [9], which are mediated by neurodevelopmental transcription factors [10]. We and others have shown previously that the transcription factor ZEB1 is a neurodevelopmental regulator of cell plasticity and tumor progression in GBM, with high ZEB1 levels promoting tumor invasion, therapy resistance and cancer stemness [11,12,13,14]. Counterintuitively, transcriptional profiling datasets reveal a survival benefit in ZEB1-high-expressing patient subsets [15,16]. In non-CNS malignancies, ZEB1 acts as a central EMT transcription factor that represses E-cadherin and drives tumor cell invasion and metastasis. It also promotes cancer cell plasticity, stem-like properties and resistance to chemo- and radiotherapy, linking ZEB1 to therapy-resistant, aggressive tumor phenotypes [17].

Cancer stem cells are a subpopulation of tumor cells with self-renewal capacity that can sustain tumor growth and contribute to therapy resistance, a concept first clearly articulated by Weissman and colleagues in 2001 [18]. In glioblastoma, such stem-like cells have been implicated in radio- and chemoresistance, providing a framework for interpreting our findings on stemness-related pathways.

Here, we evaluate transcriptional datasets of GBM patients from The Cancer Genome Atlas (TCGA) to investigate ZEB1-associated transcriptional signatures in male and female GBM patients. We find that the survival benefit of high ZEB1 expression only manifests in female patients. Stratification of TCGA data into ZEB1-high-expressing male and female cohorts reveals sex-specific transcriptional signatures of epigenetic modification associated with ZEB1 expression. Validation of sex-specific candidate epigenetic modifiers via quantitative real-time PCR highlights new avenues of investigation into transcriptional sex bias in GBM.

## 2. Materials and Methods

### 2.1. TCGA-GBM Dataset Acquisition

Transcriptomic and clinical data for GBM were obtained from TCGA via the Genomic Data Commons Data Portal (TCGA Research Network, accessed 2026) [1,2]. The TCGA-GBM cohort comprises genomic, transcriptomic and clinical metadata for primary GBM tumors, enabling integrative analysis of gene expression and patient outcomes.

RNA sequencing data generated through transcriptome profiling were used for this study. To ensure biological consistency and minimize treatment-related confounding, only samples derived from primary tumor tissue were included. In total, 370 GBM patient samples were retained for downstream analyses. Expression data consisted of 60,660 measured genes across all samples.

All data processing, statistical analyses and visualizations were performed in R (version 4.5.2) using RStudio (version 2026.04.0+526).

### 2.2. Data Access and Pre-Processing

TCGA-GBM expression and clinical data were retrieved programmatically using the TCGAbiolinks package (Bioconductor v3.22) [19]. Data were imported as a RangedSummarizedExperiment object, integrating RNA-Seq assays with corresponding patient metadata [20,21,22].

Normalized FPKM (fragments per kilobase of transcript per million mapped reads) values from unstranded RNA-Seq libraries were used as input for downstream analysis. Genes with minimal expression were filtered to improve statistical power and reduce multiple testing burden. Low-count genes were removed using standard count-based filtering procedures implemented in the genefilter and edgeR frameworks [23,24].

Non-numeric entries were removed using the dplyr package, and data were reshaped into long format using the tidyverse suite to facilitate modeling and visualization [25].

### 2.3. Stratification by ZEB1 Expression and Sex

ZEB1 expression values were extracted from the processed expression matrix. Patients were stratified into ZEB1-high- and ZEB1-low-expression groups based on the median ZEB1 expression value, resulting in two equally sized cohorts. Median-based stratification was selected to maximize statistical power and avoid bias introduced by arbitrary thresholds.

ZEB1 expression status was added to the patient metadata as a categorical variable (“high” or “low”).

Patients were subsequently grouped into a 2 × 2 factorial design based on ZEB1 expression (high/low) and biological sex (male/female), generating four experimental groups: high-male, high-female, low-male, and low-female.

### 2.4. Differential Gene Expression Analysis

Differential gene expression analysis (DEA) was performed using the limma–voom pipeline implemented in the limma package [26]. Following count normalization, the voom transformation was applied to model the mean–variance relationship of RNA-Seq data and generate precision weights suitable for linear modeling [27].

A cell-means design matrix was constructed using the formula:

design = model.matrix(~0 + group)

where group represented the four ZEB1–sex categories. This approach allowed direct estimation of group-wise expression levels without an intercept term.

*p*-values were adjusted for multiple hypothesis testing using the Benjamini–Hochberg false discovery rate (FDR) method [28]. Genes with adjusted *p*-value < 0.05 were considered significantly differentially expressed and retained for downstream analyses. Key statistics (gene identifiers, average expression, log_2_ fold change, raw and adjusted *p*-values) were exported for functional enrichment analysis.

Differential expression results were visualized using complementary graphical approaches. Hierarchical clustering heatmaps were generated using the pheatmap function [29], incorporating only candidate genes identified. Expression values were scaled by gene (row-wise) to visualize relative expression changes across samples. Sample annotations indicated sex and ZEB1 status.

Volcano plots were generated using the EnhancedVolcano package [30], plotting log_2_ fold change against −log_10_ adjusted *p*-values to highlight both magnitude and statistical significance of gene expression differences.

### 2.5. Functional Enrichment and Pathway Analysis

To explore the biological relevance of differentially expressed genes (DEGs), both over-representation analysis (ORA) and gene set enrichment analysis (GSEA) were performed [31,32].

Gene set databases were obtained using the genekitr package with gene identifiers being converted from Ensembl IDs to Entrez IDs using the transId function, with Ensembl version numbers trimmed to ensure compatibility with annotation databases [33].

For GSEA, genes were ranked by log_2_ fold change. ORA was performed using unranked gene lists of statistically significant DEGs. Statistical significance for enrichment analyses was defined as adjusted *p*-value < 0.05.

Pathway names were cleaned and standardized using the stringr package to improve clarity and consistency in visual outputs.

ORA results were visualized using bubble plots, displaying fold enrichment versus −log_10_(FDR), with bubble size representing gene count. To reduce visual clutter, only the top enriched pathways were displayed for selected gene sets.

### 2.6. Survival Analysis

Survival analyses were performed using TCGA-GBM data accessed via the GlioVis portal, which includes all samples with complete survival and expression information; as a result, the number of patients available for survival analysis differed slightly from the RNA-Seq cohort used for transcriptomic analyses [34]. Candidate genes identified from differential expression and enrichment analyses were queried individually using TCGA-GBM datasets. Survival curves were generated using median expression as a cut-off for high versus low expression.

Genes showing statistically significant survival differences (log-rank *p* < 0.05) were considered supportive of functional involvement in sex-specific survival outcomes observed in ZEB1-high GBM patients.

### 2.7. RNA Extraction, cDNA Synthesis and qPCR

Total RNA was extracted from cultured GBM cell lines (Upsala University, Sweden; hgcc.se) using the RNeasy Mini Kit (Qiagen, Hilden, Germany) according to the manufacturer’s protocol with on-column DNase treatment to eliminate genomic DNA contamination. RNA quality and integrity were assessed using a NanoDrop 2000 spectrophotometer (Thermo Fisher Scientific, Waltham, MA, USA), with samples exhibiting A260/A280 ratios between 1.8 and 2.1 and A260/A230 ratios >1.8 considered suitable for downstream applications. Complementary DNA (cDNA) synthesis was performed using 1000 ng of total RNA as a template with the QuantiTect Reverse Transcription Kit (Qiagen, Hilden, Germany), which integrates genomic DNA removal and reverse transcription in a streamlined two-step protocol.

Housekeeping genes for normalization were selected based on expression stability across experimental conditions and included GAPDH and ACTB. Primer validation was performed by confirming amplicon size through gel electrophoresis and by verifying single-product specificity by melt curve analysis (single peak with no primer-dimer formation). Gene-specific primers were reconstituted to a stock concentration of 10 µM in nuclease-free water, and cDNA template was diluted 1:10 in nuclease-free water prior to qPCR analysis to ensure template concentration fell within the linear range of amplification. Quantitative PCR reactions were performed in 384-well optical reaction plates (Thermo Fisher Scientific, Waltham, MA, USA) using Takyon Low ROX SYBR Master Mix Blue dTTP (Eurogentec, Seraing, Belgium). All samples were analyzed in technical triplicates and amplified on the Applied Biosystems QuantStudio 7 Flex Real-Time PCR System (Thermo Fisher Scientific, Waltham, MA, USA) using the following thermal cycling conditions: initial denaturation at 95 °C for 3 min to activate the hot-start polymerase, followed by 40 cycles of 95 °C for 3 s (denaturation) and 60 °C for 20 s, with fluorescence acquisition at the end of each extension step, followed by a melt curve analysis stage. Relative gene expression was calculated using the 2^−ΔΔCt^ method and one-way ANOVA with multiple comparisons used to test statistical significance.

### 2.8. Protein Extraction and Western Blot

For protein extraction, cells were lysed on ice using RIPA buffer. Protein concentrations were determined by Bradford assay (Bio-Rad, Watford, UK). For Western blotting, 5 to 10 µg of sample were mixed with equal volume of 2× Laemmli buffer (Bio-Rad, Watford, UK) containing ß-mercaptoethanol, and denatured at 95 °C for 5 min. Following separation on a mini-protean 4–15% Bis-Tris gel (Bio-Rad, Watford, UK), proteins were transferred onto PVDF membranes using a Mini Trans-Blot Turbo Transfer System (Bio-Rad, Watford, UK). Membranes were blocked and probed for primary (rabbit anti-ZEB1, Merck Life Science, Gillingham, UK; rabbit anti-GAPDH, Encor Biostechnology, Gainesville, FL, USA) and appropriate secondary antibodies and visualized using Clarity Western ECL substrate (Bio-Rad, Watford, UK) on a ChemiDoc MP imaging system (Bio-Rad, Watford, UK). GAPDH was used as loading control.

## 3. Results

### 3.1. ZEB1 Expression Predicts Overall Survival in Female but Not in Male GBM Patients in the TCGA Cohort

**Figure 1:**
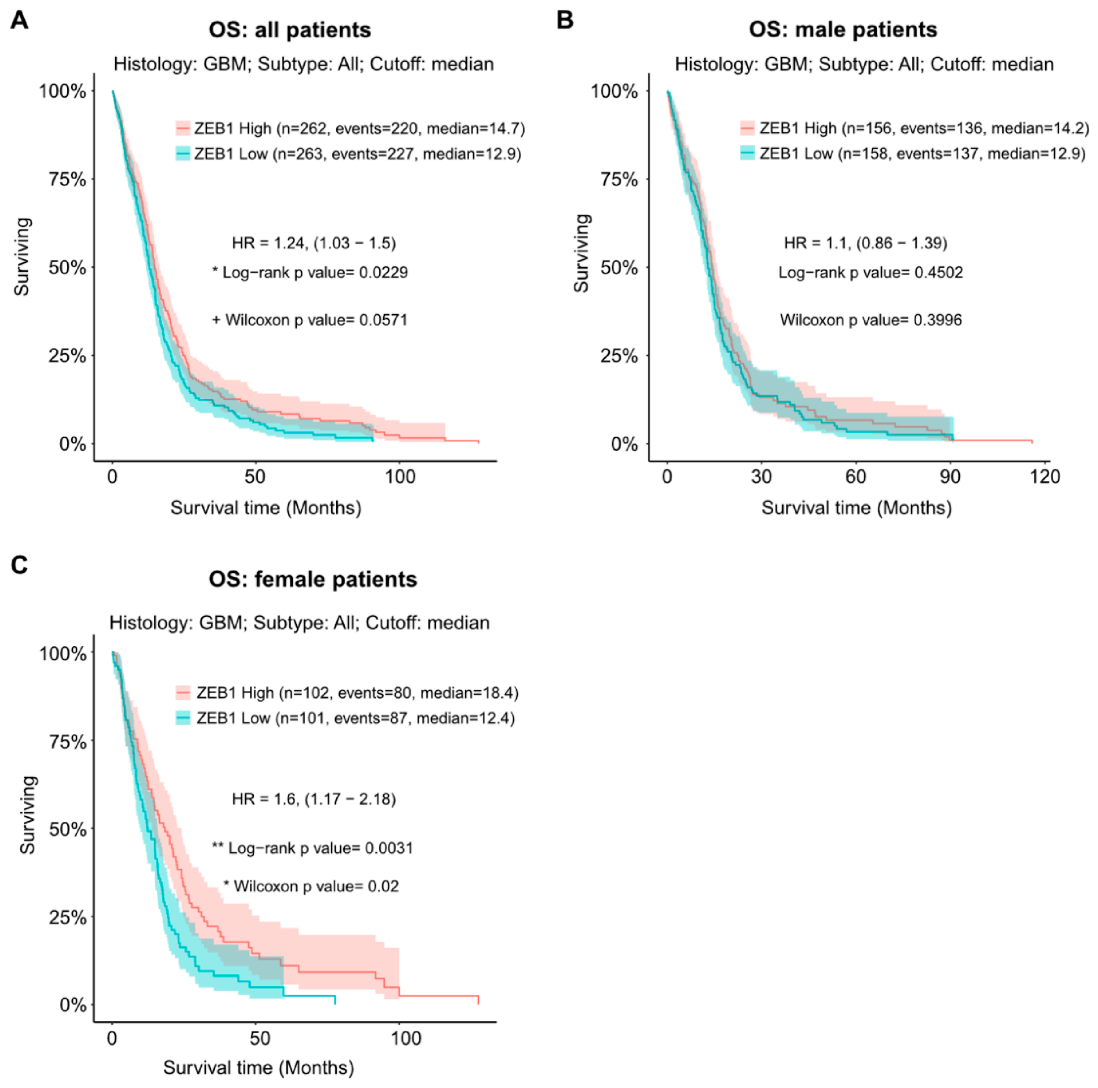
ZEB1 expression is associated with sex-specific differences in overall survival in GBM patients. (**A**) Kaplan–Meier curves show overall survival (OS) in all GBM patients stratified by median ZEB1 expression (ZEB1-high, pink; ZEB1-low, teal), with hazard ratio (HR) 1.24 (95% CI 1.03–1.5), log-rank *p* = 0.0229, and Wilcoxon *p* = 0.0571. (**B**) OS in male GBM patients stratified by median ZEB1 expression shows no significant difference between ZEB1-high and ZEB1-low groups, HR 1.1 (95% CI 0.86–1.39), log-rank *p* = 0.4502, Wilcoxon *p* = 0.3996. (**C**) OS in female GBM patients stratified by median ZEB1 expression demonstrates significantly poorer survival in the ZEB1-low group compared with the ZEB1-high group, HR 1.6 (95% CI 1.17–2.18), log-rank *p* = 0.0031, Wilcoxon *p* = 0.02. Sample sizes (*n*), number of events, and median survival (months) for each group are indicated in the legends of each panel.

Given the known sex-dependent differences in GBM incidence and outcome, we first examined whether patient sex or ZEB1 expression level were associated with overall survival in the TCGA-GBM cohort. Kaplan–Meier analysis confirmed the statistically significant difference in overall survival between high ZEB1 (*n* = 262) and low ZEB1 (*n* = 263) patients (log-rank *p* = 0.0229; Figure 1A) that was observed in previous studies [15,16]. When survival was analyzed in a sex-stratified manner, a difference emerged. In male patients, ZEB1 expression level had no bearing on overall survival (ZEB1-high male, *n* = 156; ZEB1-low male, *n* = 158; log-rank *p* = 0.4502; Figure 1B). In contrast, female patients with high ZEB1 expression demonstrated significantly prolonged overall survival compared to ZEB1-low females (ZEB1-high female, *n* = 102; ZEB1-low female, *n* = 101; log-rank *p* = 0.0031; Figure 1C). These data indicate that the prognostic association of ZEB1 expression in GBM is sex-dependent, with high ZEB1 linked to a survival advantage specifically in female patients.

**Figure 2:**
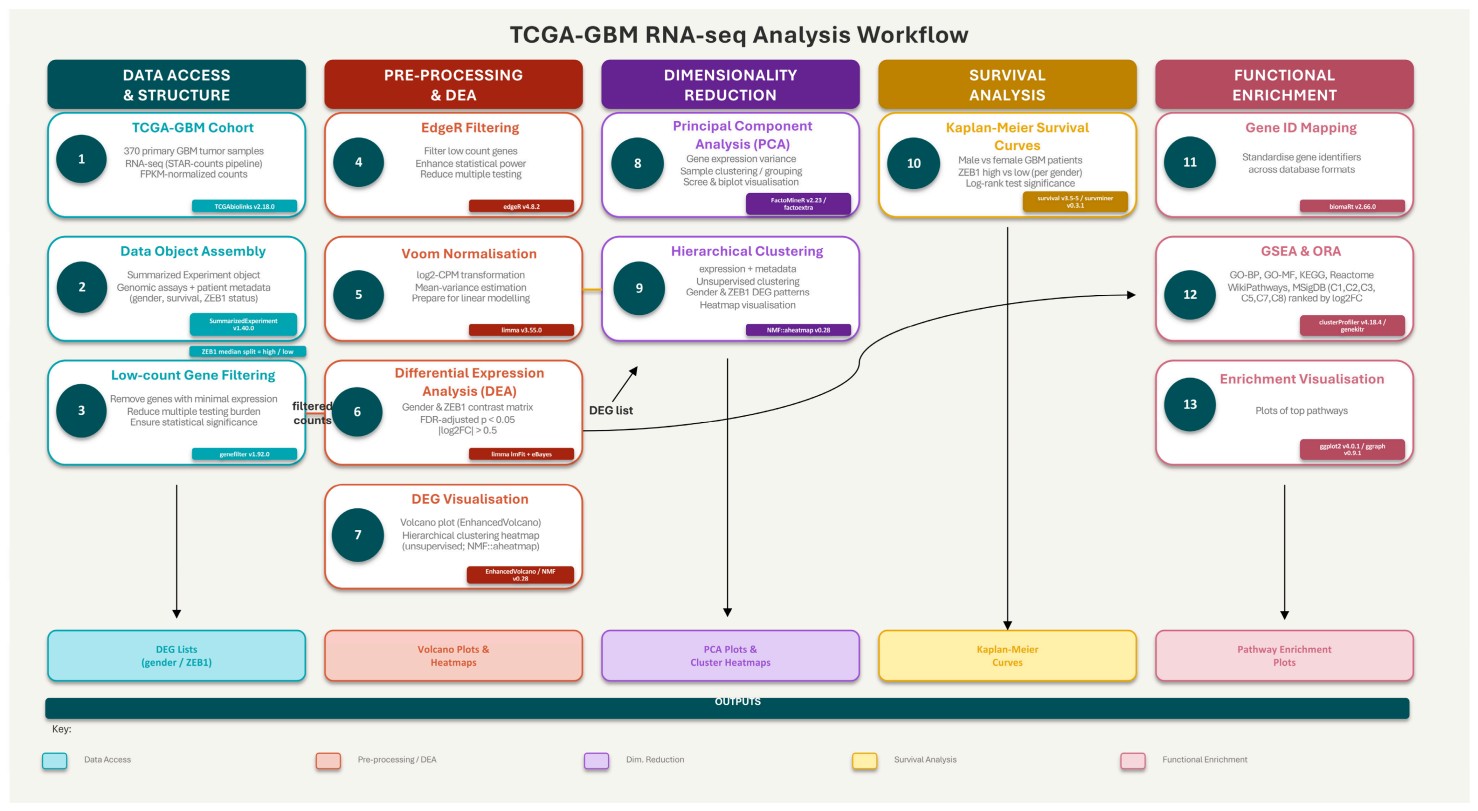
Overview of TCGA-GBM RNA-Seq analysis workflow. Schematic representation of the pipeline used to analyze RNA-Seq data from 370 TCGA GBM tumors, including data import and filtering, normalization and differential expression analysis, dimensionality reduction and clustering, Kaplan–Meier survival analysis, and downstream functional enrichment with pathway visualization. Figure was created using AI tools.

This provided the rationale for a sex-stratified transcriptomic analysis pipeline, the workflow for which is outlined in Figure 2. Briefly, the TCGA-GBM cohort was subjected to low-count gene filtering, normalization, and dimensionality reduction, followed by differential expression analysis (DEA) using EdgeR and functional enrichment via over representation analysis.

### 3.2. Sex-Stratified Transcriptomic Analysis of TCGA-GBM Cohort Identifies Candidate Genes Associated with High ZEB1 Expression

**Figure 3:**
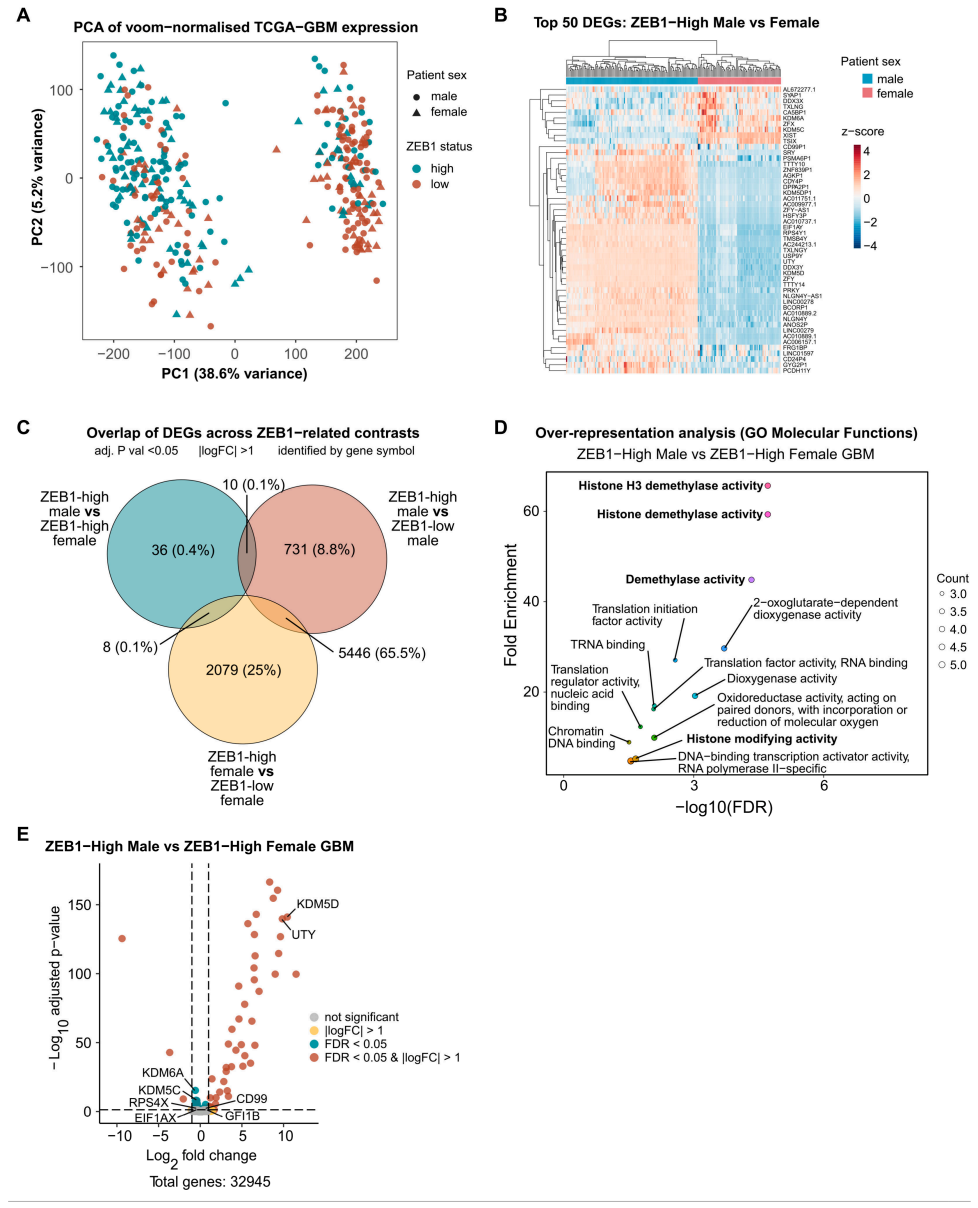
Sex- and ZEB1-associated transcriptional differences in TCGA GBM. (**A**) Principal component analysis (PCA) of voom-normalized RNA-Seq data from TCGA-GBM (*n* = 370) colored by ZEB1 status (high vs. low) and shaped by patient sex (male vs. female). (**B**) Unsupervised hierarchical clustering heatmap of the top 50 differentially expressed genes (DEGs) between ZEB1-high male and female GBM, showing z-score-scaled expression and clear segregation by sex. (**C**) Venn diagram illustrating the overlap of DEGs (protein coding only) across three ZEB1-related contrasts: ZEB1-high male vs. ZEB1-high female, ZEB1-high male vs. ZEB1-low male, and ZEB1-high female vs. ZEB1-low female. (**D**) Over-representation analysis of GO molecular function terms for DEGs between ZEB1-high male and ZEB1-high female GBM, highlighting enrichment of demethylase and histone-modifying activities. (**E**) Volcano plot of DEGs in ZEB1-high male versus ZEB1-high female GBM, indicating significantly upregulated and downregulated genes (FDR < 0.05, |log_2_FC| > 1), with selected sex chromosome-linked and chromatin-modifying genes labeled.

Principal component analysis of the voom-normalized TCGA-GBM dataset showed partial separation by patient sex and ZEB1 status, suggesting that both variables contribute to the major sources of transcriptional variance in GBM (Figure 3A). Differential expression analysis comparing ZEB1-high male tumors to ZEB1-high female GBM identified a distinct expression signature, and hierarchical clustering of the top 50 DEGs clearly segregated male and female patients, indicating robust sex-associated transcriptional differences within the ZEB1-high subgroup (Figure 3B). Across ZEB1-related contrasts, the majority of DEGs were unique to each comparison. A large set of genes was specifically altered in ZEB1-high female versus ZEB1-low female tumors, consistent with a sex-specific effect of ZEB1 on tumor transcriptional programs (Figure 3C). Over-representation analysis of DEGs between ZEB1-high male and ZEB1-high female GBM highlighted significant enrichment of GO molecular function terms linked to histone demethylase and chromatin-modifying activities, supporting the idea of sex-dependent differences in epigenetic regulation associated with ZEB1 status (Figure 3D). The corresponding volcano plot emphasized marked upregulation of Y-linked and chromatin-modifying genes (e.g., KDM5D, UTY) in ZEB1-high male tumors relative to ZEB1-high females, further supporting a model in which ZEB1 intersects with sex-chromosome-encoded epigenetic regulators to shape GBM gene expression landscapes (Figure 3E).

### 3.3. ZEB1 Regulates Candidate Gene Expression in a Sex-Dependent Manner in GBM Cells

**Figure 4:**
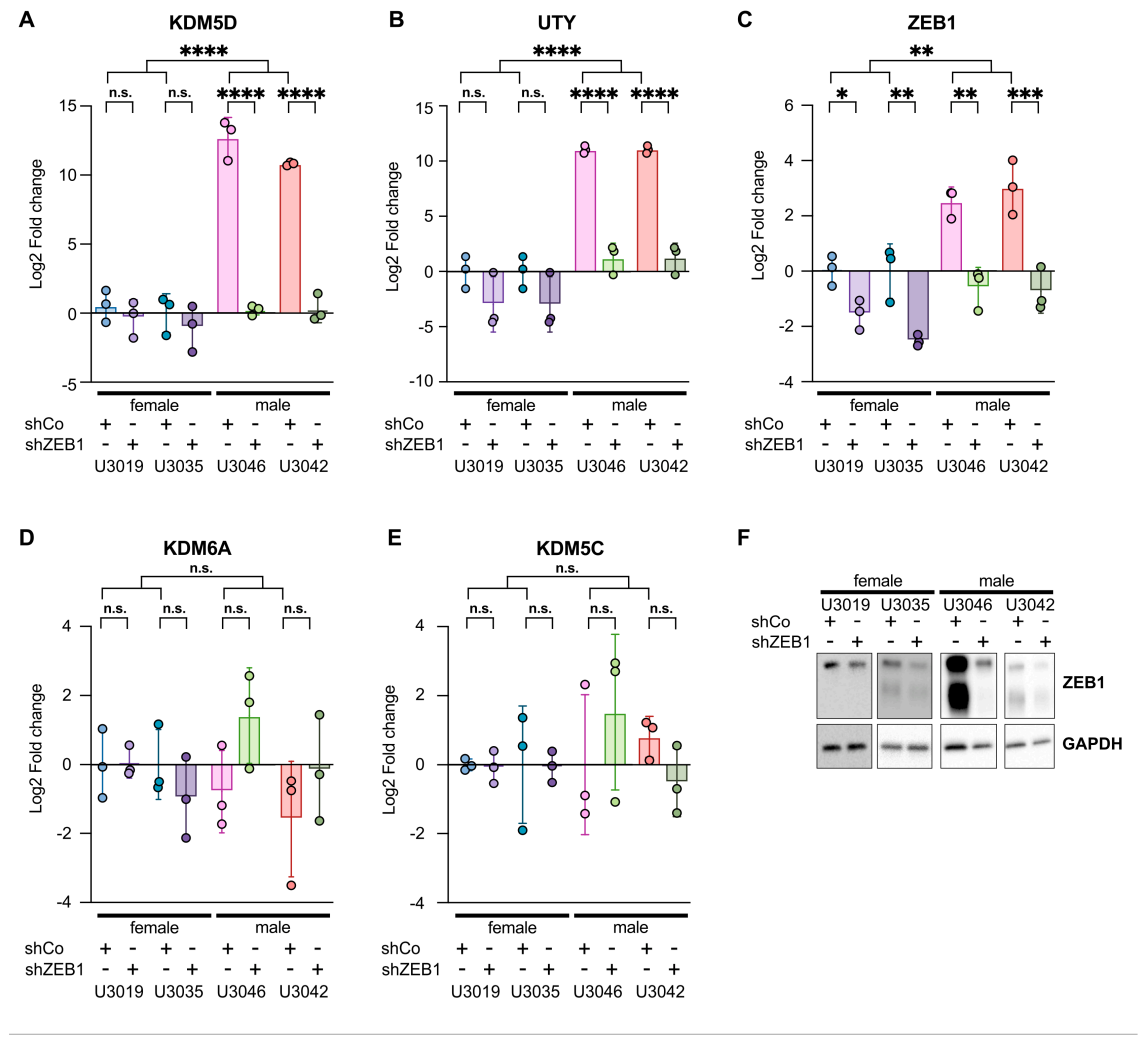
ZEB1-dependent, sex-specific regulation of Y-linked chromatin modifiers in GBM cell lines. Relative mRNA expression of KDM5D (**A**), UTY (**B**), ZEB1 (**C**), KDM6A (**D**) and KDM5C (**E**) was quantified by qPCR in two female-derived GBM lines (U3019 and U3035 shCo, (**F**)), two male-derived GBM lines (U3046 and U3042 shCo, M), and their corresponding ZEB1 knockdown derivatives (shZEB1). Expression values are presented as log_2_ fold change relative to either the female U3019 line or to the respective parental line. Bars represent mean ± SD of technical triplicates; symbols denote individual replicates. Statistical significance was assessed by one-way ANOVA with multiple comparisons; n.s., not significant; * *p* < 0.05; ** *p* < 0.01; *** *p* < 0.001; **** *p* < 0.0001. Western blot shows loss of ZEB1 protein in all shZEB1 cell lines compared to non-targeting controls (shCo, (**F**)).

We next focused on KDM5D and UTY as candidate mediators, as both were among the most significantly upregulated Y-linked chromatin modifiers in ZEB1-high male versus ZEB1-high female GBM. To validate the candidate genes identified through sex-stratified transcriptomic analysis, qPCR was performed in two female-derived (U3019, U3035) and two male-derived (U3046, U3042) GBM control cell lines (shCo), each alongside matched ZEB1 knockdown (shZEB1), with expression reported as log_2_ fold change relative to either the respective parental line or to a high female line (U3019) (Figure 4).

KDM5D expression was undetectable in the female line and remained low following ZEB1 knockdown, consistent with its Y-linked status and indicating that KDM5D is not ectopically induced by ZEB1 in female GBM cells. In contrast, KDM5D was robustly upregulated in the male-derived U3046 and U3042 cells, exhibiting an approximately 10–15-fold increase in log_2_ expression relative to the female reference, and this induction was almost completely abolished upon ZEB1 knockdown, indicating that KDM5D expression in male GBM is strongly ZEB1-dependent (Figure 4A). UTY displayed a similar pattern, with negligible expression in U3019 and U3035 shCo and shZEB1 but marked upregulation in U3046 and U3042 shCo, which was significantly reduced following ZEB1 knockdown (Figure 4B).

As expected, ZEB1 mRNA itself was significantly depleted in all shZEB1 conditions relative to their parental control lines, confirming efficient knockdown (Figure 4C). By contrast, expression of the X-linked paralogues KDM6A and KDM5C did not differ significantly between ZEB1-high and ZEB1-knockdown conditions in either sex, indicating that ZEB1 regulates the Y-linked, but not the X-linked, members of these demethylase families in this context (Figure 4D,E). Finally, we confirmed that loss of ZEB1 protein in all shZEB1 lines compared to controls by Western blot (Figure 4F).

Together, these data support KDM5D and UTY as ZEB1-regulated, male-specific chromatin modifiers and support a model in which ZEB1 cooperates with Y-linked epigenetic regulators to drive sex-biased transcriptional programs in GBM.

## 4. Discussion

Our findings indicate that ZEB1 expression carries a sex-dependent prognostic significance in GBM that may underlie the prognostic effect of ZEB1 expression when patient cohorts are analyzed without sex stratification. Whilst ZEB1 expression level conferred a significant survival difference independently across the full TCGA-GBM cohort, stratified analysis revealed a striking pattern: high ZEB1 expression was associated with significantly improved overall survival in female patients, whereas in males the effect was absent. This is consistent with emerging evidence that biological sex functions as a molecular modifier of tumor behavior rather than a demographic covariate [35,36]. GBM exhibits a male-predominant incidence of approximately 1.6:1 [37,38], yet the molecular basis of sex differences in outcome remains incompletely understood [37,39]; standard ZEB1-based prognostic strategies, evaluated predominantly in mixed-sex cohorts, may therefore systematically fail to capture clinically meaningful biology in female patients [16,40].

Transcriptomic profiling of the TCGA-GBM cohort revealed broad differential gene expression across three pairwise comparisons. Venn diagram analysis showed that the largest overlap existed between the ZEB1-high male versus female comparison and the ZEB1-high versus ZEB1-low female comparison, which is consistent with the interpretation that transcriptional divergence between ZEB1-high female tumors and their male counterparts is related to the program underpinning the female survival benefit [3,41].

The Y-chromosome-encoded candidates KDM5D and UTY were enriched in male ZEB1-high tumors, consistent with their constitutive absence from female cells [42,43,44]. qPCR validation further showed that KDM5D is selectively and robustly induced in the male-derived U3046 and U3042 lines in a ZEB1-dependent manner, while remaining undetectable in the female U3019 and U3035 lines and their ZEB1 knockdown counterparts, supporting its role as a male-specific, Y-linked effector downstream of ZEB1. KDM5D loss has been associated with immune evasion and adverse prognosis in multiple cancers [45,46,47], whilst UTY (KDM6C), the Y-linked paralogue of KDM6A, retains partial H3K27me3 demethylase activity; its relative under-expression in male GBM may contribute to chromatin compaction at tumor-suppressive loci [42,43,44]. ZEB1 knockdown in the male-derived U3046 and U3042 cell lines markedly downregulated UTY expression, suggesting that ZEB1 is a regulator of UTY expression in male tumors—reflecting ZEB1’s context-dependent transcriptional roles that merits further mechanistic dissection [15,16]. Interestingly, transcriptional analysis demonstrated that ZEB1 does not regulate expression of X-chromosomal counterparts of these candidates (KDM5C and KDM6A), raising the possibility that that divergent transcriptomes between male and female patients may be affected by differential epigenetic regulatory mechanisms that depend at least partially on ZEB1 [42,43,48].

Sex-specific divergence was most pronounced in chromatin organization terms: demethylase-related GO molecular function terms were male-enriched, whilst broader chromatin organization pathways were female-enriched, supporting the hypothesis that sex differences in chromatin regulatory capacity—mediated through sex-chromosome-encoded histone demethylases—interact with ZEB1-associated transcriptional programs to give rise to distinct tumor phenotypes [43,49,50,51].

Key limitations include the modest female subgroup size and the absence of prospective sex-stratified design in TCGA-GBM. Furthermore, very long-term survivors in our historical glioblastoma cohort (up to ~100 months) may include tumors that would now be classified as astrocytoma, IDH-mutant CNS WHO grade 4 rather than glioblastoma, IDH-wildtype, which could modestly bias our survival estimates and sex-specific analyses. It is also important to consider that women generally have longer life expectancy than men and to evaluate whether these background sex differences in non-tumor-related mortality contribute to the observed survival patterns in GBM. Future work should prioritize replication in independent cohorts, functional characterization of candidate genes through sex-matched perturbation experiments, and mechanistic interrogation of ZEB1–demethylase interactions via ChIP-seq and ATAC-seq. Single-cell RNA sequencing of sex-stratified primary specimens would allow these signatures to be resolved at the level of individual tumor subpopulations.

## 5. Conclusions

This study shows that the prognostic relevance of ZEB1 in GBM is sex-dependent, with high expression associated with a significant survival advantage exclusively in female patients. Transcriptomic analysis identified sex-biased candidate genes—including the Y-linked regulators KDM5D and UTY—whose differential expression in ZEB1-high tumors is consistent with the chromatin regulatory asymmetry conferred by sex chromosome gene dosage. Pathway analysis revealed shared ZEB1-associated translational programs alongside sex-divergent chromatin organization pathways, consistent with this mechanistic framework. These findings underscore the importance of sex as an analytical variable in GBM biomarker research and highlight sex-chromosomally encoded epigenetic regulators as a plausible mechanistic basis for ZEB1’s sex-dependent clinical behavior.

## Data Availability

The original data presented in the study are openly available at https://portal.gdc.cancer.gov/projects/TCGA-GBM (accessed on 12 February 2026) and were accessed using the TCGAbiolinks package (v2.40.0).
